# Therapeutic silencing of fat-specific protein 27 improves glycemic control in mouse models of obesity and insulin resistance[S]

**DOI:** 10.1194/jlr.M069799

**Published:** 2016-12-29

**Authors:** Cédric Langhi, Noemí Arias, Ananthi Rajamoorthi, Jeannine Basta, Richard G. Lee, Ángel Baldán

**Affiliations:** Edward A. Doisy Department of Biochemistry & Molecular Biology* Saint Louis University, Saint Louis, MO 63104; Department of Internal Medicine,† Saint Louis University, Saint Louis, MO 63104; Cardiovascular Group, Antisense Drug Discovery,§ Ionis Pharmaceuticals, Carlsbad, CA 92010; Center for Cardiovascular Research** Saint Louis University, Saint Louis, MO 63104; Liver Center,†† Saint Louis University, Saint Louis, MO 63104

**Keywords:** cell death-inducing DFFA-like effector C, diabetes, fatty liver, steatosis, antisense therapy

## Abstract

Obesity is a component of the metabolic syndrome, mechanistically linked to diabetes, fatty liver disease, and cardiovascular disease. Proteins that regulate the metabolic fate of intracellular lipid droplets are potential therapeutic candidates to treat obesity and its related consequences. *CIDEC* (cell death-inducing DFFA-like effector C), also known in mice as *Fsp27* (fat-specific protein 27), is a lipid droplet-associated protein that prevents lipid mobilization and promotes intracellular lipid storage. The consequences of complete loss of FSP27 on hepatic metabolism and on insulin resistance are controversial, as both healthy and deleterious lipodystrophic phenotypes have been reported in *Fsp27*^−/−^ mice. To test whether therapeutic silencing of *Fsp27* might be useful to improve obesity, fatty liver, and glycemic control, we used antisense oligonucleotides (ASOs) in both nutritional (high-fat diet) and genetic (leptin-deficient *ob/ob*) mouse models of obesity, hyperglycemia, and hepatosteatosis. We show that partial silencing *Fsp27* in either model results in the robust decrease in visceral fat, improved insulin sensitivity and whole-body glycemic control, and tissue-specific changes in transcripts controlling lipid oxidation and synthesis. These data suggest that partial reduction of FSP27 activity (e.g., using ASOs) might be exploited therapeutically in insulin-resistant obese or overweight patients.

Overweight and obesity affect ∼70% and ∼30% of Americans, respectively (1, 2). The excessive accumulation of triacylglyceride (TAG)-rich lipid droplets (LDs) in adipose tissue, liver, and muscle is linked to multiorgan impaired insulin sensitivity, diabetes, atherogenic dyslipidemia, and nonalcoholic fatty liver disease (2, 3). Importantly, visceral (abdominal) obesity and impaired glycemic control are core drivers of the metabolic syndrome and increase the risk of cardiovascular disease (4–6). Obesity is at its core a disorder of energy homeostasis, due to the imbalance between caloric intake, storage, and expenditure. Proteins at the surface of intracellular LDs determine the metabolic fate of the stored lipids (7, 8), and they are likely pharmacological candidates to manage obese patients. Cell death-inducing DFFA-like effector C (*CIDEC*)/fat-specific protein 27 (*Fsp27*) promotes the fusion of smaller LDs and acts as a lipolytic barrier (9–11) and is abundant in white adipose tissue (WAT) and brown adipose tissue (BAT) (12–15). Hepatic *Fsp27* expression is very low, but rises during fasting (16, 17) and in the fatty livers of mice (17–19) and obese patients (20, 21). Overexpression of *Fsp27* increases LD formation and TAG contents in cultured cells (12, 13, 18). Conversely, acute knockdown of *Fsp27* results in smaller, multilocular LDs in cultured adipocytes (12, 13), abrogates fasting-induced hepatic TAG accumulation (17), and ameliorates hepatosteatosis in both *ob/ob* mice (19) and mice fed an HFD (17). Consistently, both *Fsp27*^−/−^ and *ob/ob*×*Fsp27*^−/−^ mice are resistant to diet-induced obesity, dyslipidemia, and hepatosteatosis and have improved insulin sensitivity (14, 15). A recent report on hepatocyte-specific *Fsp27*^−/−^ mice suggests that loss of FSP27 ameliorates alcohol-induced steatohepatitis in mice fed a liquid ethanol diet (which also provides 30 kcal% from fat) (22). Based on these studies, it was initially speculated that reduction of FSP27 activity might be exploited therapeutically to benefit obese patients. However, when kept under long-term caloric intake surplus, *Fsp27*^−/−^ (23), *ob/ob*×*Fsp27*^−/−^ (23), and adipocyte-specific *Fsp27*^−/−^ (24) mice develop fatty liver, insulin resistance, and dyslipidemia. This latter phenotype is consistent with a report of a patient carrying a homozygous nonsense mutation in *CIDEC* who also developed partial lipodystrophy, hepatosteatosis, and insulin-resistant diabetes (25). The reasons for the discrepancies between the short- and long-term consequences of loss of FSP27 activity on hepatic and whole-body lipid metabolism and insulin sensitivity under overnutrition conditions remain to be fully elucidated.

While genetic loss-of-function animal models are useful to identify the role of genes on particular metabolic pathways, they are not necessarily informative on the value of therapeutic interventions that only partially decrease the amounts or activity of those same genes. Here we tested the consequences of therapeutic silencing of *Fsp27* using generation 2.0 antisense oligonucleotides (ASOs) in both a dietary [high-fat diet (HFD)] and a genetic (leptin-deficient *ob/ob*) model of obesity, hepatosteatosis, and insulin resistance (26–28). The proposal that long-term reduction of *CIDEC/Fsp27* improves obesity and/or insulin sensitivity, under conditions of overnutrition, has never been tested. We hypothesized that residual FSP27 activity in adipose tissue and liver would prevent the deleterious consequences noted in long-term HFD-fed whole-body and adipocyte-specific *Fsp27*^−/−^ mice (23, 24). Our data show that silencing *Fsp27* results in the robust decrease in visceral fat, modest “browning” of white adipocytes, tissue-specific changes in transcripts controlling lipid oxidation and/or synthesis, and, importantly, improved insulin sensitivity and whole-body glycemic control, with no apparent adverse effects on hepatic steatosis. These data strongly suggest that reduction of FSP27 activity (e.g., using ASO) might be therapeutically beneficial to insulin-resistant obese or overweight patients.

## EXPERIMENTAL PROCEDURES

### Chemicals

Chimeric 2’methoxyethyl control (5′-CCTTCCCTGAAGGTT­CCTCC) and anti-Fsp27 (5′-CAGACTCTAATACCATTCAC) oligonucleotides were synthesized and purified as described (29), suspended in saline, and stored at –20°C until used. Glucose and sodium pyruvate were purchased from Sigma (St. Louis, MO). A prescription for Humulin-R (Eli Lilly, Indianapolis, IN) was filled by a veterinarian at Saint Louis University.

### Mouse studies

**Fig. 1.**
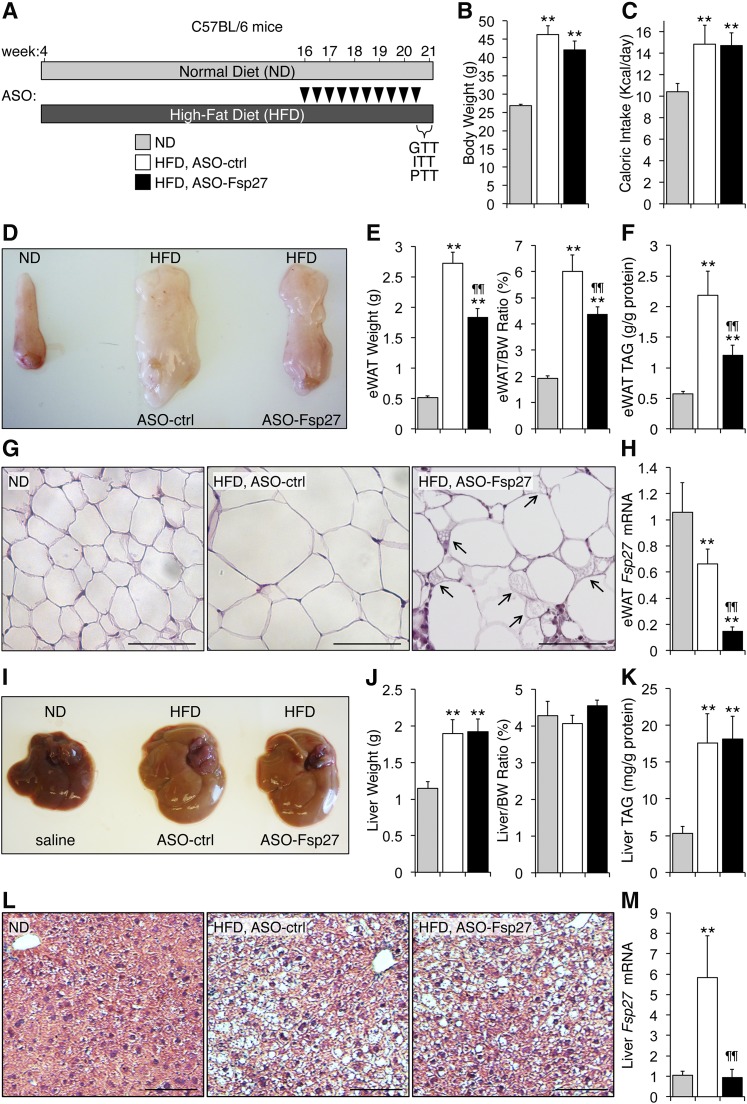
ASO-Fsp27 treatment reduces visceral adiposity without worsening hepatic steatosis in HFD-fed mice. A: Male C57BL/6 mice (4 weeks old, n = 6) were fed an HFD for 17 weeks, where the last five included treatment with ASO-ctrl or ASO-Fsp27 (25 mg/kg ip twice weekly; arrowheads). An age-matched control group of mice (n = 4) was kept on an ND. B: Body weight at time of euthanization. C: Food intake during the last 2 weeks. D: Representative macroscopic appearance of the left eWAT pad. E: Absolute and relative weight of eWAT. F: eWAT triacylglyceride (TAG) contents. G: Multilocular LDs (arrows) were recognizable only in ASO-Fsp27 eWAT sections stained with hematoxylin and eosin. Scale bar represents 100 μm. H: Relative eWAT *Fsp27* mRNA expression. I: Representative macroscopic appearance of the livers. J: Absolute and relative liver weight. K: Hepatic TAG contents. L: Paraffin-embedded sections of livers stained with hematoxylin and eosin show HFD-induced cell ballooning and steatosis. Scale bar represents 100 μm. M: Relative hepatic *Fsp27* mRNA expression. Data are shown as mean ± SEM. * *P* ≤ 0.05 and ** *P* ≤ 0.01, HFD vs. ND; ^¶¶^ *P* ≤ 0.01, ASO-Fsp27 vs. ASO-ctrl. These data are representative of two independent experiments.

**Fig. 2.**
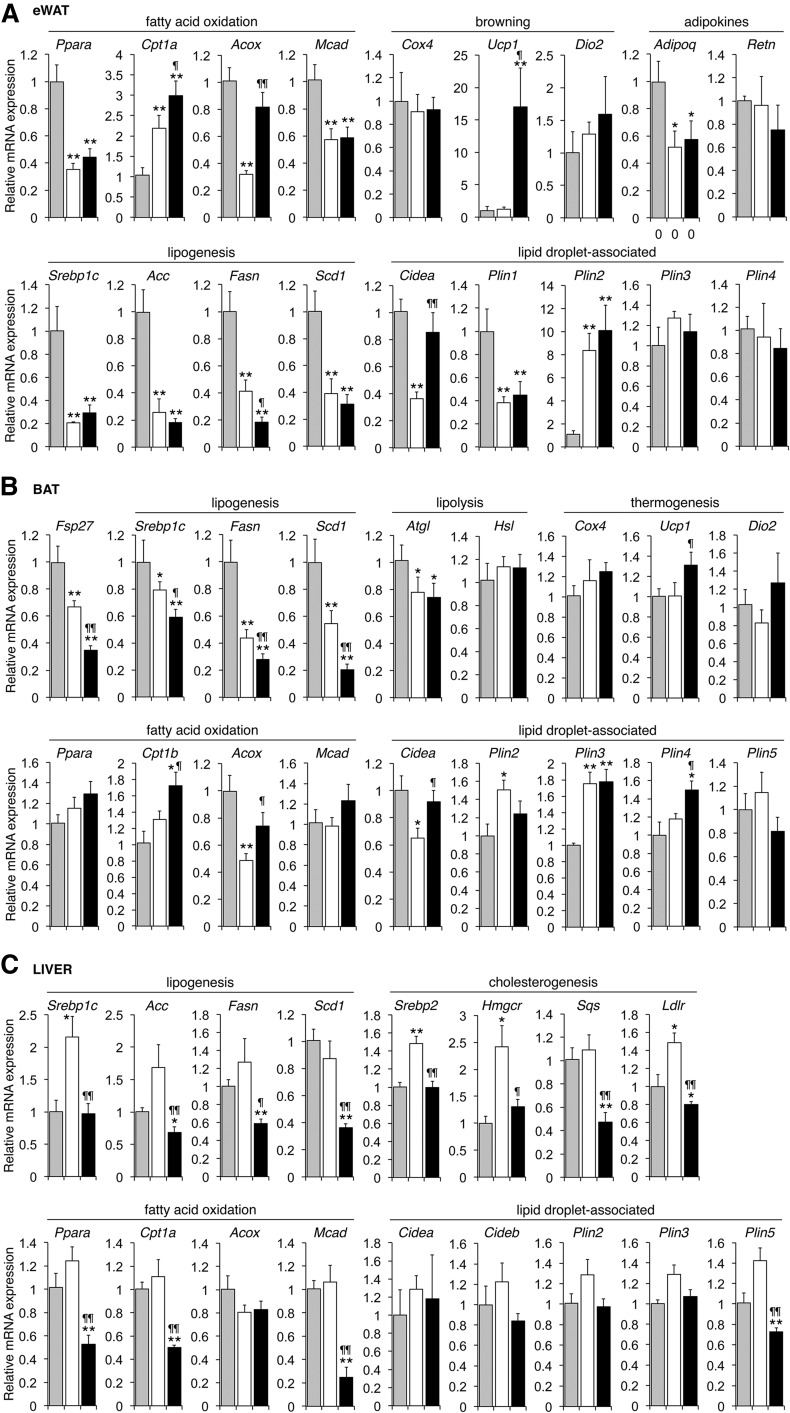
Tissue-specific changes in transcripts related to lipid metabolism in HFD-fed mice treated with ASO-Fsp27. Relative expression in eWAT (A), scapular BAT (B), and liver (C) of selected transcripts from mice shown in Fig. 1, fed ND (gray bars) or HFD plus treatment with ASO-ctrl or ASO-Fsp27 (white and black bars, respectively). Data are shown as mean ± SEM (n = 6). * *P* ≤ 0.05 and ** *P* ≤ 0.01, HFD vs. ND. ^¶^ *P* ≤ 0.05 and ^¶¶^ *P* ≤ 0.01, ASO-Fsp27 vs. ASO-ctrl. These data are representative of two independent experiments.

**Fig. 3.**
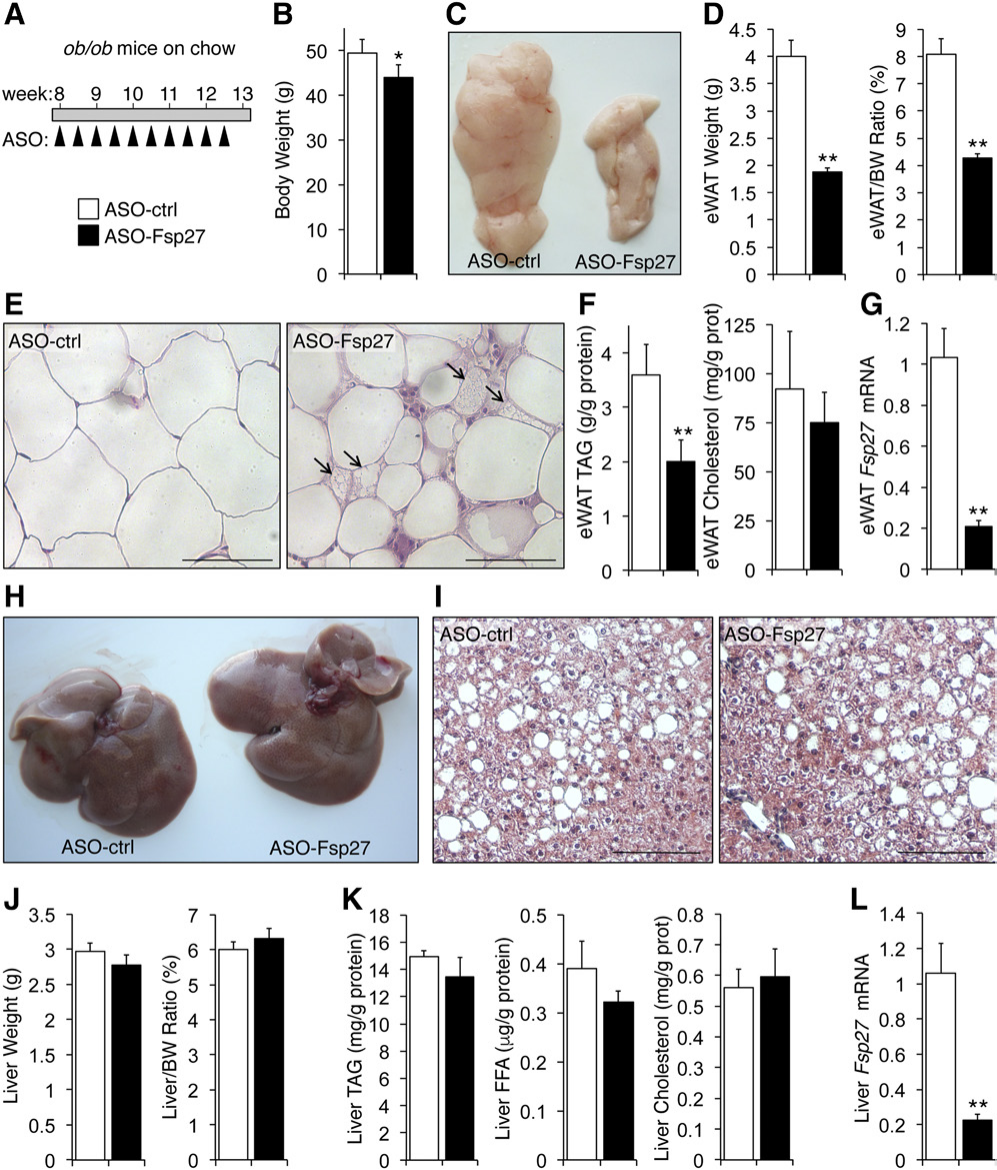
Reduced epidydimal fat without changes in liver steatosis in genetically obese *ob/ob* mice treated with ASO-Fsp27. A: Chow-fed animals (n = 6) were treated as shown. B: Body weight at time of euthanization. C: Representative macroscopic appearance of the left eWAT pad. D: Absolute and relative weight of eWAT. E: Multilocular LDs (arrows) were recognizable only in ASO-Fsp27 eWAT sections stained with hematoxylin and eosin. Scale bar represents 100 μm. F: Triacylglyceride (TAG) and cholesterol contents in eWAT. G: Relative eWAT *Fsp27* mRNA expression. H: Representative macroscopic appearance of the livers. I: Hepatosteatosis was evident in paraffin-embedded sections of livers stained with hematoxylin and eosin, showing HFD-induced cell ballooning and steatosis. Scale bar represents 100 μm. J: Absolute and relative liver weight. K: Hepatic TAG, unesterified fatty acids (FFA), and cholesterol contents. L: Relative hepatic *Fsp27* mRNA expression. Overall, the phenotypic changes in these mice mimic the results obtained in HFD-fed C57BL/6 mice (Fig. 1). Data are shown as mean ± SEM. * *P* ≤ 0.05 and ** *P* ≤ 0.01, ASO-Fsp27 vs. ASO-ctrl. Scale bars in histology pictures are 100 μm. These data are representative of two independent experiments.

**Fig. 4.**
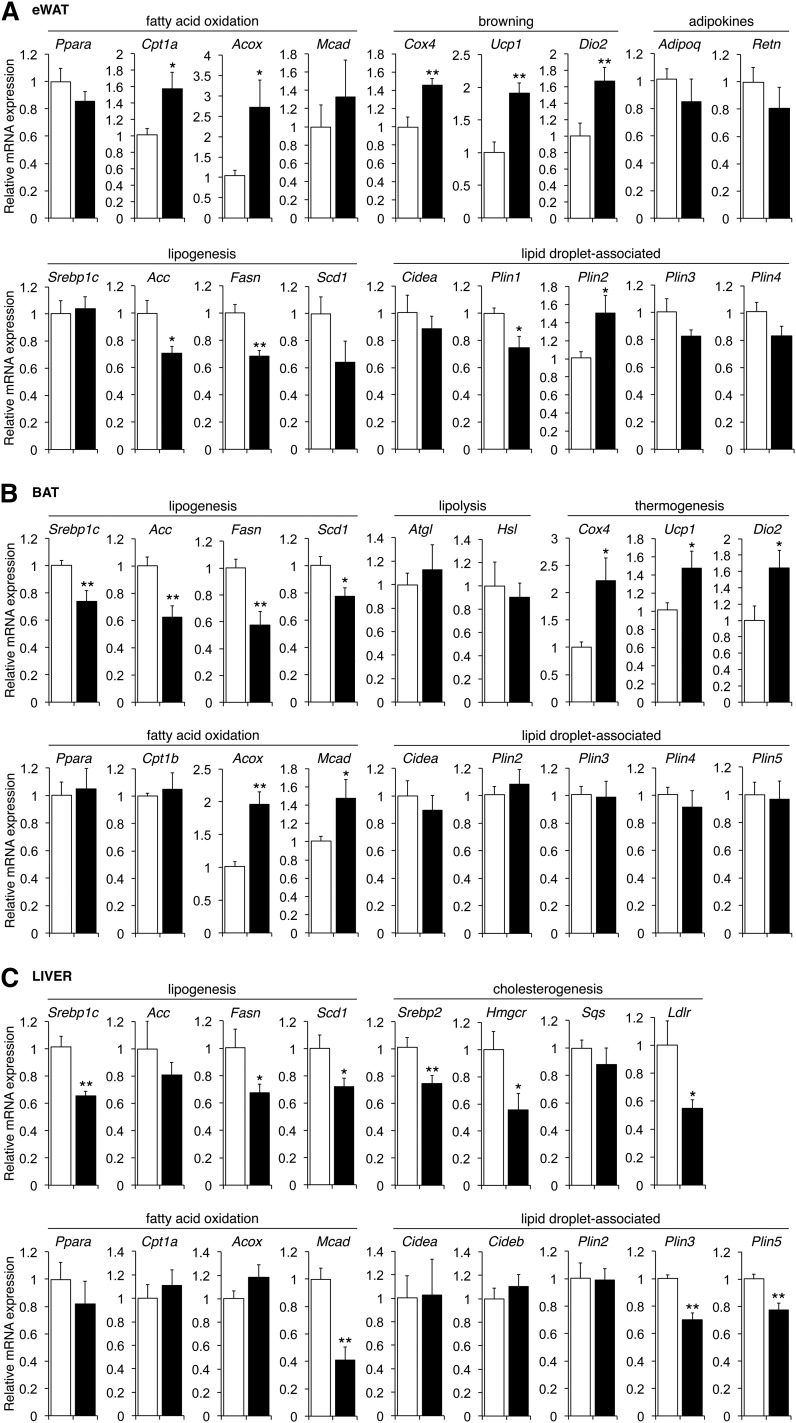
Tissue-specific changes in transcripts related to lipid metabolism in *ob/ob* mice treated with ASO-Fsp27. Relative expression of selected transcripts related to lipid metabolism in eWAT (A), liver (B), and BAT (C) in *ob/ob* mice treated with ASO-ctrl and ASO-Fsp27 shown in Fig. 3. Data are shown as mean ± SEM. (n = 6). * *P* ≤ 0.05 and ** *P* ≤ 0.01, ASO-Fsp27 vs. ASO-ctrl. These data are representative of two independent experiments.

All animals were maintained in a 12 h/12 h light/dark cycle with ad libitum access to food and water. Male C57BL/6 mice (Jackson Laboratories stock 000664) were fed normal diet (ND; PicoLab Rodent Diet 20) or an HFD (TestDiet 58Y1 providing 60 Kcal% from fat). Male *ob/ob* mice (Jackson Laboratories stock 000632) were kept on standard chow. Where indicated, ASOs were injected ip at 25 mg/kg twice weekly (Monday and Thursday) for 5 weeks. See **Fig. 1A** and Fig. 3A for details on timelines. Robust silencing of *Fsp27* was noted in liver, epididymal white adipose tissue (eWAT), and scapular BAT (**Figs. 1**, **2**, **3**, **4**). Animal studies were conducted in conformity with the Public Health Service policy on humane care and use of laboratory animals, and approved by the IACUC at Saint Louis University.

### Glucose, insulin, and pyruvate challenges

GTTs were performed in mice fasted overnight and then injected ip with 2 g/kg glucose. ITTs were performed in mice fasted for 4 h and then injected ip with 0.75 U/kg Humulin-R. PTTs were performed in mice fasted overnight and then injected ip with 2 g/kg sodium pyruvate. In all studies, glycemia was monitored at the indicated time points from tail bleeds using a Contour glucometer (Bayer, Pittsburgh, PA).

### In vivo insulin signaling

Studies were performed as described (30–32). After ND or HFD feedings and ASO treatments as shown in Fig. 1A, mice were fasted overnight, then injected ip with 100 μl saline or 5 U/kg Humulin-R. Exactly 10 min after injection, mice were euthanized, and liver, eWAT, and gastrocnemius muscle biopsies were flash-frozen in liquid nitrogen before protein extraction. Fifty micrograms of protein were resolved in precasted 4–12% Bis–Tris gels (Life Technologies, Carlsbad, CA), transferred to polyvinylidene difluoride membranes, and probed with antibodies for protein kinase B (AKT) (Cell Signaling 9272; 1:1,000 dilution; Danvers, MA), phospho-AKT (Cell Signaling 9271; 1:1,000 dilution), insulin receptor (IR) (Santa Cruz sc-711; 1:1,000 dilution; Dallas, TX), phospho-IR (Cell Signaling 3024; 1:1,000 dilution), and β-actin (Thermo RB-9421; 1:1,000 dilution; Carlsbad, CA), as described (30–32), in TBS-Tween20 containing 5% nonfat dry milk. Immune complexes were detected with horseradish peroxidase-conjugated secondary antibodies [Bio-Rad 170-6515 (anti-rabbit), 170-6516 (anti-mouse); 1:5,000 dilution; Hercules, CA], using a high-resolution PXi-4 imager (SynGene, Frederick, MD). Band intensities were calculated using GeneTools version 4.03 software (SynGene).

### Histology

Samples of livers, eWAT, and BAT were fixed in 10% formalin, postfixed in 50% ethanol, and embedded in paraffin blocks. Sections (4 μm) were processed for hematoxylin and eosin staining using standard techniques. For immunostaining of F4/80^+^ macrophages, adipose sections were boiled for antigen retrieval in 10 mM citric acid, pH 6, and endogenous peroxidases blocked with 3% hydrogen peroxide. Sections were incubated with 10% goat serum for 20 min at room temperature and avidin/biotin blocking solution (Vector Laboratories, Burlingame, CA). Sections were incubated overnight at 4°C with rat anti-F4/80, clone CI:A3-1 (Bio-Rad MCA497GA; 1:150 dilution; Raleigh, NC) in 1% BSA. Antibody reactivity was detected using biotin labeled anti-rat IgG (1:100 dilution; Jackson ImmunoResearch, West Grove PA), HRP streptavidin (1:100, Invitrogen), and DAB substrate kit (ThermoFischer, Walthman, MA). Sections were counterstained with hematoxylin.

### Lipid analysis

Tissue lipids were extracted into CHCl_3_ by a modified Folch method (33), solubilized in water, and quantitated enzymatically using kits for triglycerides, total cholesterol, and FFAs (Wako Chemicals, Richmond, VA). Results were normalized to protein.

### Plasma analysis

Circulating lipids were quantitated enzymatically in 5 μl plasma using Wako kits. Activities of alanine aminotransferase (ALT) and aspartate aminotransferase (AST) were quantified enzymatically using colorimetric kits (BioVision K753-100, Milpitas, CA). Plasma insulin was measured using an ELISA kit (Millipore EZRMI-13K, Billerica, MA).

### RNA analysis

RNA was isolated from tissues using Direct-zol RNA miniprep kit (ZYMO Research, Irvine, CA), and analyzed by real-time quantitative PCR using PowerSybrGreen (Life Technologies, Carlsbad, CA) and a LightCycler LC480 instrument (Roche, Indianapolis, IN). Values were normalized to *36b4*, and relative expression calculated using the ΔΔC_T_ method. Primer sets are shown in supplemental Table S1.

### Enzymatic activity assays

Tissues (∼200 mg) were homogenized in 150 mM KCl, 1 mM MgCl_2_, 10 mM N-acetyl-cysteine, 0.5 mM dithiothreitol, pH 7.6, and ultracentrifuged at 100,000 *g* for 40 min at 4°C. FASN activity was determined in the supernatants, as described (34), and expressed as nmol of NADPH consumed/min/mg of protein. In parallel experiments, tissues (∼200 mg) were homogenized in 250 mM sucrose, 1 mM EDTA, 10 mM Tris-HCl, pH 7.4, and centrifuged at 700 *g* for 10 min at 4°C. The supernatants were then centrifuged at 12,000 *g* for 10 min at 4°C. The pellets (containing mitochondrial and peroxisomal fractions) were resuspended in 70 mM sucrose, 220 mM mannitol, 1 mM EDTA, 2 mM HEPES, pH 7.4, and used to assay CPT1 enzymatic activity as described (35), and expressed as nmol of CoA released/min/mg of protein.

### Statistics

Data are shown as mean ± SEM. Differences between groups were analyzed by one-way ANOVA, or two-way ANOVA followed by post hoc Bonferroni’s test, using SPSS version 20.0 (IBM, Armonk, NY). Differences were considered significant at *P* ≤ 0.05.

## RESULTS

### Therapeutic silencing of FSP27 in HFD-fed mice reduces visceral adiposity without worsening hepatosteatosis

We fed C57BL/6 mice an HFD (60 kcal% fat) for 17 weeks, where the last 5 weeks included treatment with ASO-ctrl or ASO-Fsp27; an age-matched group was kept on ND (Fig. 1A). As expected, mice on HFD gained more weight than those on ND (Fig. 1B). Caloric intake was elevated in the HFD-fed mice, compared with ND, but was not altered by ASO-Fsp27 treatment, compared with ASO-ctrl (Fig. 1C). Analysis of plasma lipids revealed a significant drop in plasma TAG in ASO-Fsp27 mice, compared with ASO-ctrl (**Table 1**). The mass of eWAT and eWAT TAG contents were reduced by 30% and 40%, respectively, in HFD-fed mice treated with ASO-Fsp27, compared with ASO-ctrl (Fig. 1D–F). As expected, adipocytes were enlarged in HFD-fed mice, compared with ND (Fig. 1G). Adipocytes in ASO-Fsp27-treated mice appeared heterogeneous in size, and ∼5% appeared multilocular (suggestive of modest “browning” of the tissue), compared with ASO-ctrl (Fig. 1G). Interestingly, treatment with ASO-Fsp27 also resulted in the appearance of crownlike structures (Fig. 1G). Immunohistochemical analysis using an F4/80 antibody identified these as macrophages (supplemental Fig. S1A), although the mRNA expression of typical M1-like macrophage inflammatory mediators (*Cd11c*, *Tnfα*,* Il6*) was found to be unchanged in ASO-Fsp27, compared with ASO-ctrl, eWAT samples (supplemental Fig. S1B). ASO-Fsp27 effectively silenced the expression of adipose *Fsp27* (Fig. 1H) and resulted in the induction of both oxidative genes (*Cpt1α*,* Acox*) and the BAT marker *Ucp1* (again consistent with “browning”), compared with ASO-ctrl (Fig. 2A). Enzymatic activity assays in the same eWAT samples confirmed the increased CPT1 enzymatic activity (supplemental Fig. S2A). Collectively, these data suggest that *Fsp27* silencing promotes lipid utilization in visceral WAT and decreases adiposity and are reminiscent of *Fsp27*^−/−^ mice that show strong browning of white adipocytes (14, 15, 24). Similar to eWAT, reduced expression of *Fsp27* in scapular BAT lead to increased levels of oxidative genes (*Cpt1β*,* Acox*) but also reduced levels of lipogenic genes [sterol regulatory element-binding protein 1c (*Srebp1c*),* Fasn*,* Scd1*], compared with ASO-ctrl (Fig. 2B).

**TABLE 1.** Plasma parameters in C57BL/6 mice treated with ASO-ctrl or ASO-Fsp27

Diet Treatment	ND	HFD ASO-ctrl	HFD ASO-Fsp27
N	4	6	6
Triglycerides*^a^* (mg/dl)	54.4 ± 2.6	58.0 ± 8.2	35.1 ± 2.6*^b,c^*
Cholesterol*^a^* (mg/dl)	104.2 ± 17.2	189.8 ± 5.8*^d^*	207.9 ± 8.7*^d^*
FFA*^a^* (mM)	0.15 ± 0.02	0.19 ± 0.02	0.20 ± 0.02
Glucose*^a^* (mg/dl)	126.0 ± 5.1	170.4 ± 4.2*^d^*	156.0 ± 4.7*^d^*
Glucose*^e^* (mg/dl)	57.3 ± 4.6	112.4 ± 2.7*^d^*	96.6 ± 6.8*^d^*
Insulin*^a^* (ng/ml)	0.75 ± 0.32	3.09 ± 0.88*^b^*	1.91 ± 0.71*^b^*
AST*^a^* (mU/ml)	13.8 ± 1.0	17.3 ± 1.5	13.9 ± 0.9
ALT*^a^* (mU/ml)	0.69 ± 0.02	1.11 ± 0.14*^b^*	1.19 ± 0.14*^b^*

Mice were fed ND or HFD and treated with ASOs as shown in Fig. 1A. Data are expressed as mean ± SEM.

a Six hours food removal.

b *P* ≤ 0.05 HFD vs. ND.

c *P* ≤ 0.05 ASO-Fsp27 vs. ASO-ctrl.

d *P* ≤ 0.01 HFD vs. ND.

e Fasted overnight.

Data in Fig. 1 also show that, as expected, HFD increased hepatic mass and TAG accumulation and promoted mild liver damage, compared with ND (Fig. 1I–L; AST/ALT levels in Table 1). Notably, and contrary to long-term HFD-fed *Fsp27*^−/−^ mice (23, 24), treatment with ASO-Fsp27 did not change liver appearance or TAG contents (Fig. 1I–L). Effective silencing of hepatic *Fsp27* (Fig. 1M) resulted in reduced expression of both lipogenic (*Srebp1c*,* Fasn*,* Acc*,* Sdc1*) and, unexpectedly, oxidative (*Ppara*, *Cpt1a*,* Mcad*) genes, compared with ASO-ctrl (Fig. 2C). Results from enzymatic activity assays for hepatic FASN and CPT1 (supplemental Fig. S2B) were consistent with these mRNA data.

**Fig. 5.**
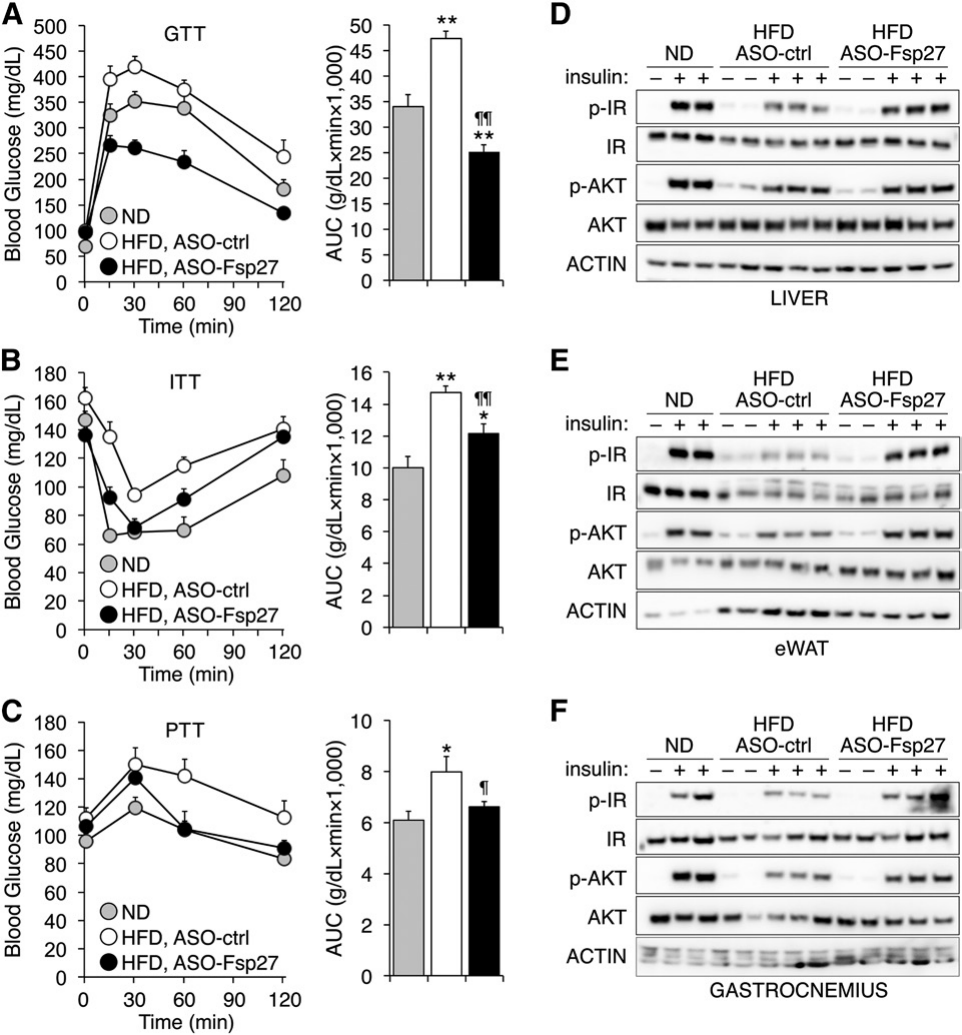
Improved whole-body glycemic control and insulin sensitivity in mice treated with ASO-Fsp27. Male C57BL/6 mice were treated as in Fig. 1A. A–C: GTT, ITT, and PTT were performed as described in Experimental Procedures. AUC, area under the curve. Data are shown as mean ± SEM (n = 8). * *P* ≤ 0.05 and ** *P* ≤ 0.01, HFD vs. ND; ^¶^ *P* ≤ 0.05 and ^¶¶^ *P* ≤ 0.01, ASO-Fsp27 vs. ASO-ctrl. D–F: Immunoblots for total and phosphorylated IR and AKT in the liver, eWAT, and gastrocnemius muscle from an independent group of C57BL/6 mice treated as above, then fasted overnight, injected ip with saline or 5 U/kg insulin, and euthanized exactly 10 min later. Quantification of signal intensities is provided in supplemental Fig. S4.

An independent experiment in a separate cohort of mice (n = 6/group) replicated the data shown in Figs. 1 and (data not shown for mice in **Fig. 5** below). Importantly, these results suggest that systemic therapeutic silencing of *Fsp27*, although not effective in reducing hepatosteatosis, might not lead to a deleterious liver phenotype in this model of dietary obesity, as reported in HFD-fed mice after complete loss of FSP27 activity (23, 24).

### Therapeutic silencing of FSP27 in *ob/ob* mice reduces visceral adiposity without worsening hepatosteatosis

The consequences of therapeutic *Fsp27* silencing were notoriously similar in genetically obese mice. Chow-fed *ob/ob* mice (n = 6) were dosed with control or anti-Fsp27 ASOs for 5 weeks (Fig. 3A). Body weight was modestly reduced in mice injected with ASO-Fsp27, compared with ASO-ctrl (Fig. 3B). No effects were noted on food intake or plasma lipids (**Table 2**). The eWAT mass was reduced by ∼50% in the same mice, both in absolute and relative terms (Fig. 3C, D). Histological analysis revealed small and medium-sized adipocytes (∼10% with multilocular LDs) in ASO-Fsp27-treated mice (Fig. 3E). Consistently, TAG contents were significantly reduced in these samples, compared with ASO-ctrl (Fig. 3F). Macrophage-containing crownlike structures were also identified in eWAT sections from ASO-Fsp27-treated mice (Fig. 3E). Analysis of selected transcripts in the same eWAT samples showed the efficient transduction with the ASO (Fig. 3G), and the induction of both oxidative (*Cpt1α*,* Acox*) and BAT specific (*Cox4*,* Ucp1*,* Dio2*) genes in ASO-Fsp27 mice, compared with ASO-ctrl (Fig. 4A). A modest increase in perilipin-2 was noted in the same samples (Fig. 4A). Together, these data are consistent with those in HFD-fed mice above, and suggest accelerated lipid catabolism, without changes in lipogenesis, in WAT following *Fsp27* silencing. Data in Fig. 4B show that ASO-Fsp27 was also effective in silencing *Fsp27* in scapular BAT and resulted in reduced expression of lipogenic genes in ASO-Fsp27-treated mice, compared with ASO-ctrl.

**TABLE 2.** Physiologic parameters in ob/ob mice treated with ASO-ctrl or ASO-Fsp27

	ASO-ctrl	ASO-Fsp27
N	6	6
Food intake (Kcal/day)	18.3 ± 0.4	17.4 ± 1.3
Plasma TAG*^a^* (mg/dl)	58.3 ± 5.6	53.4 ± 1.8
Plasma cholesterol*^a^* (mg/dl)	189.4 ± 8.9	182.9 ± 7.8
Plasma FFA*^a^* (mM)	0.25 ± 0.02	0.24 ± 0.03
Plasma glucose*^a^* (mg/dl)	256.5 ± 29.5	248.5 ± 15.4
Plasma glucose*^b^* (mg/dl)	101.6 ± 6.6	84.0 ± 3.9*^c^*
Plasma insulin*^a^* (ng/ml)	11.6 ± 1.2	11.5 ± 1.5
Plasma AST*^a^* (mU/ml)	25.7 ± 3.1	26.9 ± 2.6
Plasma ALT*^a^* (mU/ml)	0.38 ± 0.08	0.43 ± 0.05

Mice were fed chow and treated as shown in Fig. 3A. Data are expressed as mean ± SEM.

a Six hours food removal.

b Fasted overnight.

c *P* ≤ 0.05.

Despite the profound phenotype in white adipose tissue, the 5-week treatment with ASO-Fsp27 in *ob/ob* mice again did not lead to changes in liver macroscopic appearance (Fig. 3H), histology (Fig. 3I), absolute or relative tissue weight (Fig. 3J), or lipid contents (Fig. 3K), compared with ASO-ctrl. All livers appeared enlarged and steatotic, and liver damage was apparent from the modestly elevated plasma AST levels (Table 2). The ASO effectively silenced hepatic *Fsp27* expression (Fig. 3L). No changes were noted in the lipid oxidation transcriptional program (Fig. 4C), but the livers of ASO-Fsp27-treated mice showed reduced expression of both lipogenic (*Srebp1c* targets) and cholesterogenic (*Srebp2* targets) genes (Fig. 4C). An independent experiment in a separate cohort of *ob/ob* mice (n = 6/group) replicated the data shown in Figs. 3 and (data not shown). Results from FASN and CPT1 enzymatic activity assays in adipose tissue (supplemental Fig. S3A) and liver (supplemental Fig. S3B) were consistent with the mRNA data. These results are also consistent with the data in HFD-fed mice above and again suggest that systemic therapeutic silencing of *Fsp27* does not lead to the deleterious hepatic phenotype noted after genetic loss of *Fsp27* and overnutrition (23, 24).

### Improved glycemic control in mice treated with anti-Fsp27 ASOs

Given the striking effect of ASO-Fsp27 treatment on the mass of eWAT, and the importance of visceral adiposity on whole-body glycemic control, we speculated that an improvement in insulin sensitivity might follow therapeutic silencing of FSP27. The reports on the consequences of complete loss of FSP27 activity on glycemic control in *Fsp27*^−/−^ mice are conflicting, and authors showed both beneficial (14, 15) and detrimental (23, 24) phenotypes. However, no data are available on the impact of therapeutic silencing (e.g., partial loss) of FSP27 on glycemic control. To test whether reduced FSP27 activity improves insulin sensitivity in one or more tissues, a new group of C57BL/6 mice were fed HFD and injected with ASOs, or kept on ND, as in Fig. 1A. We favored this model instead of leptin-deficiency, because HFD is a more physiologic model of metabolic syndrome and thus more relevant to human disease. Glycemic control was determined following glucose, insulin, or pyruvate overload (Fig. 5A–C). Data show that *Fsp27* silencing indeed improved glycemic control. Unexpectedly, glucose tolerance in HFD-fed, ASO-Fsp27-treated mice exceeded that of ND-fed mice (Fig. 5A). Finally, to determine whether ASO-Fsp27 increases insulin sensitivity in liver, adipose tissue, and/or muscle, a third group of C57BL/6 mice were fed HFD and injected with ASOs, or kept on ND, as in Fig. 1A. After 5 weeks of treatment, mice were fasted overnight and then injected with saline or 5 U/kg insulin. Liver, eWAT, and gastrocnemius muscle were dissected exactly 10 min later and used to assess insulin sensitivity by measuring the phosphorylation of both the IR and AKT (Fig. 5D–F; supplemental Fig. S4). As expected, insulin promoted the strong phosphorylation of both IR and AKT in all tissues in mice fed an ND. Consistent with HFD-induced insulin resistance, insulin-dependent phosphorylation of IR and AKT was decreased in HFD-fed, ASO-ctrl-treated mice, compared with ND. Treatment with ASO-Fsp27, however, improved/rescued insulin-dependent phosphorylation of IR and AKT in liver, eWAT, and (to a lesser degree) muscle. Collectively, our data suggest that therapeutic silencing of *Fsp27* may be useful to not only decrease visceral obesity, but also to improve whole-body insulin sensitivity and glucose metabolism.

## DISCUSSION

Dysregulation of lipid metabolism is the basis of common medical disorders in Western populations, such as cardiovascular disease, hyperlipidemia, obesity, and insulin resistance. This is the first report on the consequences of therapeutic intervention on *Fsp27* on obesity, fatty liver, and glycemic control. Longer-term studies will be necessary to confirm whether the phenotypic changes reported herein are sustained over time, but our data provide in vivo evidence that supports the therapeutic potential of reducing *Fsp27* expression/activity in obese patients to reduce visceral adiposity and multiorgan insulin resistance. From a basic science perspective, our results also highlight the important differences between genetic and intervention animal models.

The data presented here are partially in conflict with previous reports using whole-body or adipocyte-specific *Fsp27*^−/−^ mice, which showed detrimental effects on glycemic control after long-term overnutrition (23, 24). These different outcomes in knockout and ASO studies are not irreconcilable, though. We propose that residual FSP27 activity in WAT and liver in ASO-treated mice, independent of dietary fat overload, accounts for the disparities. Critically, although ASO-treated mice show a substantial reduction in adipose mass, they do not develop severe lipodystrophy like *Fsp27*^−/−^ animals (14, 15, 23, 24). Similar to other models of lipodystrophy (36, 37), the unregulated lipolysis in adipose tissue and sustained delivery of fatty acids to the liver likely leads to accelerated hepatic reesterification and storage, exceeding the ability of hepatocytes to cope with the lipid surplus, and ultimately resulting in a deleterious phenotype (fatty liver, insulin resistance) in of *Fsp27*^−/−^ mice. In contrast, our data show that therapeutic knockdown of *Fsp27* reduces visceral fat and improves whole-body glycemic control, without leading to further adverse effects in liver (hepatosteatosis was similar to that in ASO-ctrl-treated mice). Interestingly, F4/80^+^ crownlike structures were noticeably more abundant in eWAT from ASO-Fsp27-treated mice, compared with ASO-ctrl. These structures are a typical histologic hallmark of rapidly expanding visceral fat depots of both HFD-fed mice and obese patients (38, 39). Importantly, macrophages are also rapidly recruited into the adipose tissue under conditions of accelerated lipolysis during active weight loss (40, 41); this lipolysis-driven recruitment, however, is not correlated with a significant change in the expression of typical inflammatory markers such as *Tnfa*,* Il-6*, or *Cd11c* (40). Our data in the eWAT of ASO-Fsp27-treated mice nicely recapitulates these latter studies and provides further support for the idea of accelerated lipid mobilization in WAT following FSP27 silencing.

In our study, both the genetic and the nutritional models responded similarly to ASO-Fsp27 intervention. A curious aspect of our studies was the distinctive metabolic adaptations of different tissues following partial loss of FSP27. Hence, in the white adipose tissue the treatment induced the transcriptional program controlling lipid oxidation (PPARα targets and thermogenic genes) in both HFD-fed wild-type and chow-fed *ob/ob* mice, while also reducing the transcriptional program controlling de novo lipogenesis (SREBP1c targets) in chow-fed *ob/ob* mice. The livers of the same ASO-Fsp27-treated mice, however, did not increase the lipid oxidative program (in HFD-fed mice PPARα targets were actually reduced), but instead repressed both the lipogenic and cholesterogenic transcriptional programs (SREBP1c and SREBP2 targets, respectively), compared with ASO-ctrl treatment. Importantly, determination of FASN and CPT1 enzymatic activities in the same eWAT and livers confirmed the different metabolic adaptations of these tissues in response to ASO-Fsp27 treatment. Finally, the BAT of ASO-Fsp27-treated mice reduced the lipogenic transcriptional program and increased the fatty acid oxidation transcriptional program, compared with ASO-ctrl, in both animal models. Despite the abundant transcriptional changes, TAG contents did not change in the livers of ASO-Fsp27-treated mice, compared with ASO-control-treated animals. We hypothesize that the sharp reduction of visceral fat pads in the former mice results in accelerated delivery of fatty acids to the liver that overcomes the effects of decreased hepatic de novo lipogenesis. But why are hepatic lipids not mobilized toward oxidation, like in WAT? The decreased mRNA expression of PPARα targets in the livers of ASO-Fsp27-treated animals is perplexing, given that these same transcripts are elevated in the adipose tissues of the same mice. Previous studies in cells suggested that FSP27 activity prevents the mobilization of LD-contained lipids toward the mitochondria for oxidation (9), and we reported that loss of FSP27 activity via a shRNA accelerated the turnover of TAG in mouse primary hepatocytes (17), likely increasing their availability of substrates for mitochondrial β-oxidation. Interestingly, a decrease in PPARα targets was also described in the livers of HFD-fed *Fsp27*^−/−^ mice (23). We hypothesize that hepatic FSP27 activity controls the accumulation/release of an endogenous PPARα ligand. This proposal is consistent with studies showing that hepatic de novo lipogenesis generates a PPARα ligand that is accumulated in the hepatocyte (presumably in LDs) for subsequent release into circulation (42). Future studies will determine whether silencing of Fsp27 with ASO alters the synthesis and/or circulating levels of such endogenous PPARα ligands. Nevertheless, in an acute (7-day) intervention study we showed that combining Wy14643 (a synthetic PPARα agonist) with an adenovirus-mediated shRNA against *Fsp27* normalized hepatic TAG levels in C57BL/6 mice previously fed an HFD for 8 weeks, compared with fibrate or shRNA alone (17). The decrease in PPARα targets in both long-term HFD-fed *Fsp27*^−/−^ mice (23) and ASO-treated mice (herein) suggest that a combination of ASO plus a PPARα synthetic agonist might be necessary to stimulate the efficient mobilization and oxidation of hepatic lipids, and to provide therapeutic reduction of hepatic steatosis. Future studies in our laboratory will test this hypothesis.

Visceral fat and liver play central roles on whole-body insulin sensitivity and glucose metabolism (43, 44) and are core drivers of the metabolic syndrome and cardiovascular risk. Obesity and fatty liver disease are intimately connected and pose a severe public health burden, given their high and growing prevalence in both adult and pediatric populations, and the risk they confer for disability and mortality. The toolbox to manage overweight and obese patients is scarce, beyond lifestyle modifications, appetite suppressant drugs, and major surgical procedures. Better medical interventions are sorely needed for these patients. The data presented herein highlight the therapeutic potential of anti-*Fsp27* ASOs to manage tissue lipid storage/utilization and to improve glycemic control in these patients.

## Supplementary Material

Supplemental Data
