# Supplementary material for: Therapeutic silencing of fat-specific protein 27 improves glycemic control in mouse models of obesity and insulin resistance
Source: J Lipid Res. 2016 Dec 29;58(1):81–91. doi: 10.1194/jlr.M069799 (PMC5234712; doi:10.1194/jlr.M069799)
Supplement: Supplemental Data [file supp_58_1_81__index.html]

Therapeutic silencing of fat-specific protein 27 improves glycemic control in mouse models of obesity and insulin resistance — Supplemental Data 

# Therapeutic silencing of fat-specific protein 27 improves glycemic control in mouse models of obesity and insulin resistance

## Supplemental Data

- Supplemental information (.pdf, 1.1 MB) - Supplemental information
