# Supplementary material for: Therapeutic silencing of fat-specific protein 27 improves glycemic control in mouse models of obesity and insulin resistance
Source: J Lipid Res. 2016 Dec 29;58(1):81–91. doi: 10.1194/jlr.M069799 (PMC5234712; doi:10.1194/jlr.M069799)
Supplement: Supplemental Data [file 10.1194_M069799_jlr.M069799-1.pdf]

Supplemental Information

# Therapeutic silencing of *Fsp27* improves glycemic control in mouse models of obesity and insulin resistance

Cédric Langhi[1], Noemí Arias[1], Ananthi Rajamoorthi[1], Jeannine Basta[2], Richard G. Lee[3], and Ángel Baldán[1,4,5]

[1]Edward A. Doisy Department of Biochemistry & Molecular Biology, Saint Louis University, Saint Louis, MO 63104; [2]Department of Internal Medicine, Saint Louis University, Saint Louis, MO 63104; [3]Cardiovascular Group, Antisense Drug Discovery, Ionis Pharmaceuticals, Carlsbad, CA 92010; [4]Center for Cardiovascular Research, and [5]Liver Center, Saint Louis University, Saint Louis, MO 63104

Supplemental Figures S1, S2, S3, S4
Supplemental Table S1

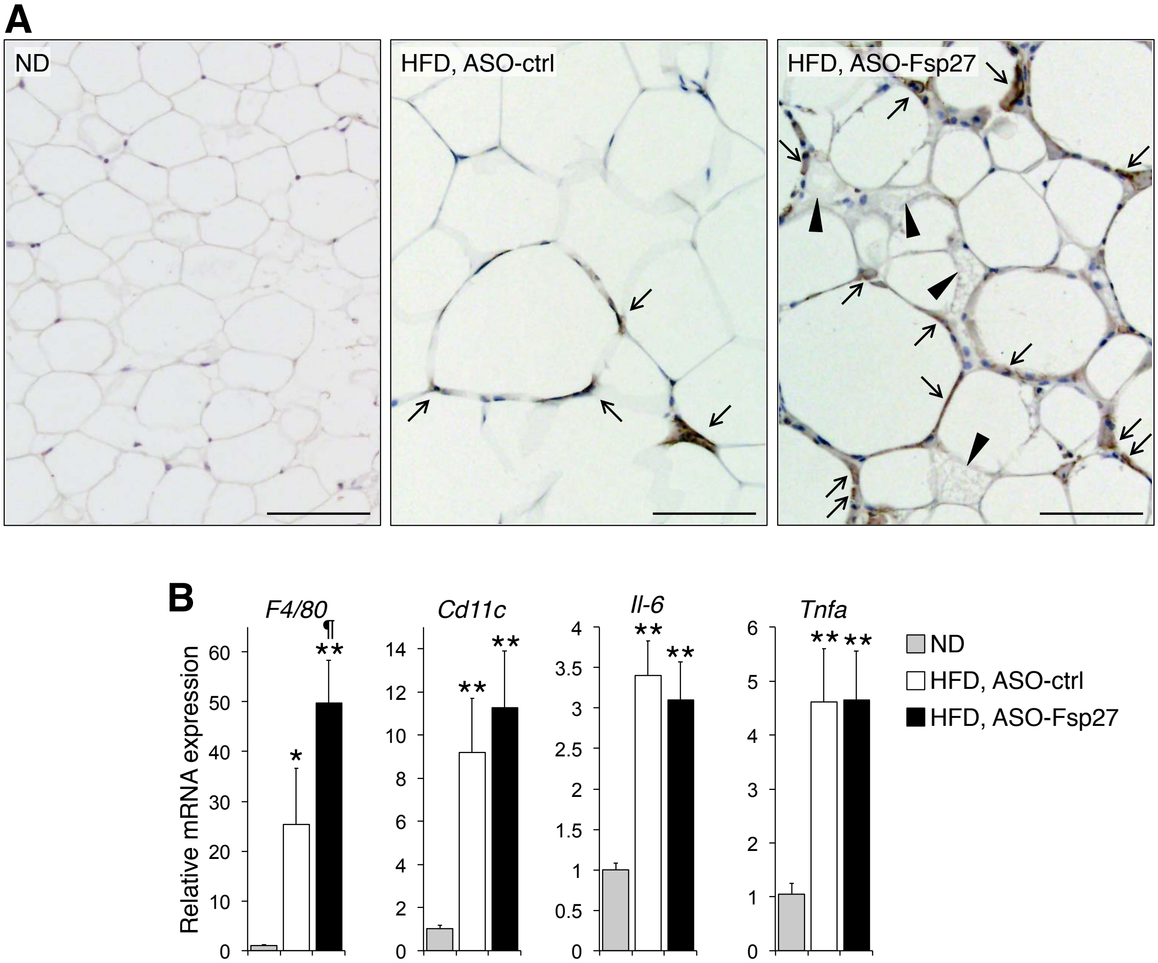


**Supplemental Figure S1.** Increased infiltration of macrophages into the eWAT of ASO-Fsp27 treated mice. C57BL/6 mice were fed normal diet (ND; gray bars), or high-fat diet (HFD) plus treatment with ASO-ctrl or ASO-Fsp27 (white and black bars, respectively), as shown in Fig. 1A. (A) Representative immunohistochemistry micrographs of tissue sections using an F4/80 antibody. Brown precipitate is noted in infiltrating macrophages (arrows). Multilocular adipocytes (arrowheads) were noted only in ASO-Fsp27 treated mice. (B) Relative mRNA expression of selected markers associated to pro-inflammatory M1-like macrophages. Data are shown as mean ± s.e.m (n=6). *$P \leq 0.05$ and **$P \leq 0.01$, HFD *vs.* ND. ¶$P \leq 0.05$, ASO-Fsp27 *vs.* ASO-ctrl.

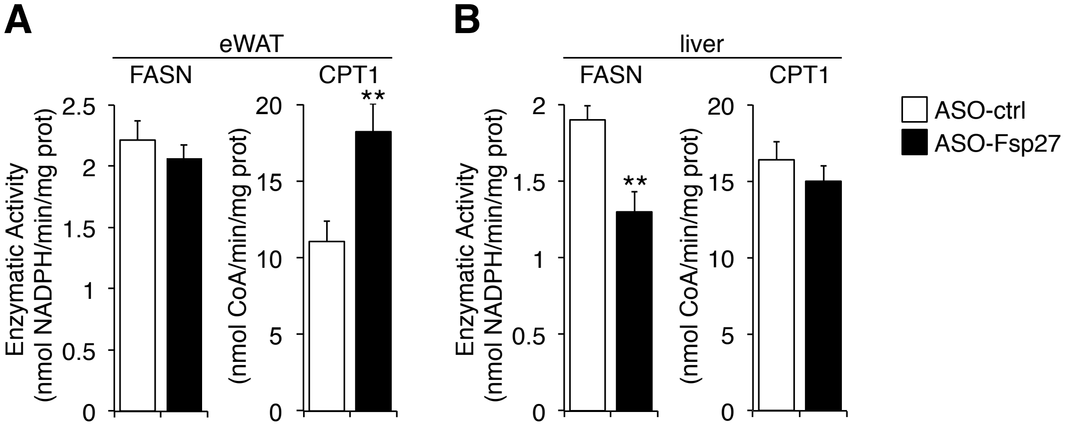


**Supplemental Figure S2.** Enzymatic activities of FASN and CPT1 in eWAT and liver in HFD-fed C57BL/6 mice treated with ASO-FSp27. Animals were treated with ASO-ctrl or ASO-Fsp27, as shown in Fig. 1A. Enzymatic activities in extracts from eWAT (A) and liver (B) were determined as described in methods. Data are shown as mean ± s.e.m (n=6). $^{**}P \leq 0.01$, ASO-Fsp27 *vs.* ASO-ctrl.

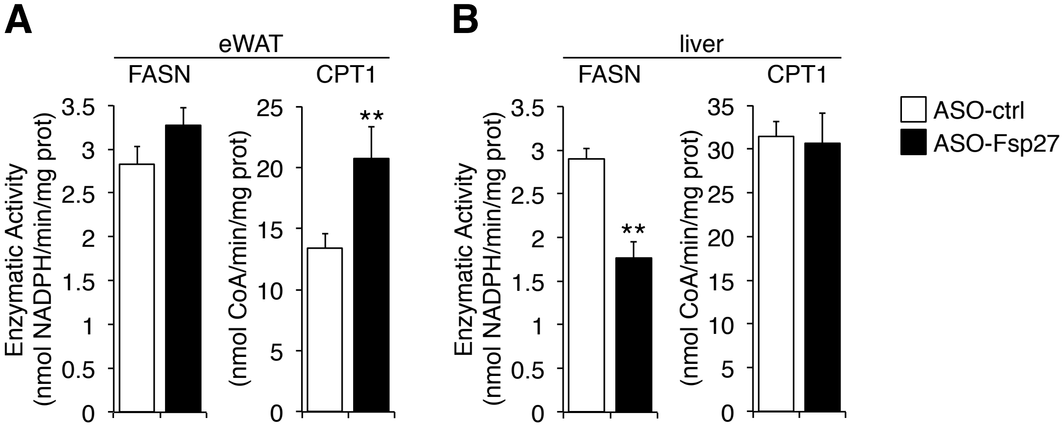


**Supplemental Figure S3.** Enzymatic activities of FASN and CPT1 in eWAT and liver in chow-fed *ob/ob* mice treated with ASO-FSp27. Animals were treated with ASO-ctrl or ASO-Fsp27, as shown in Fig. 3A. Enzymatic activities in extracts from eWAT (A) and liver (B) were determined as described in methods. Data are shown as mean ± s.e.m (n=6). ***P*≤0.01, ASO-Fsp27 *vs.* ASO-ctrl.

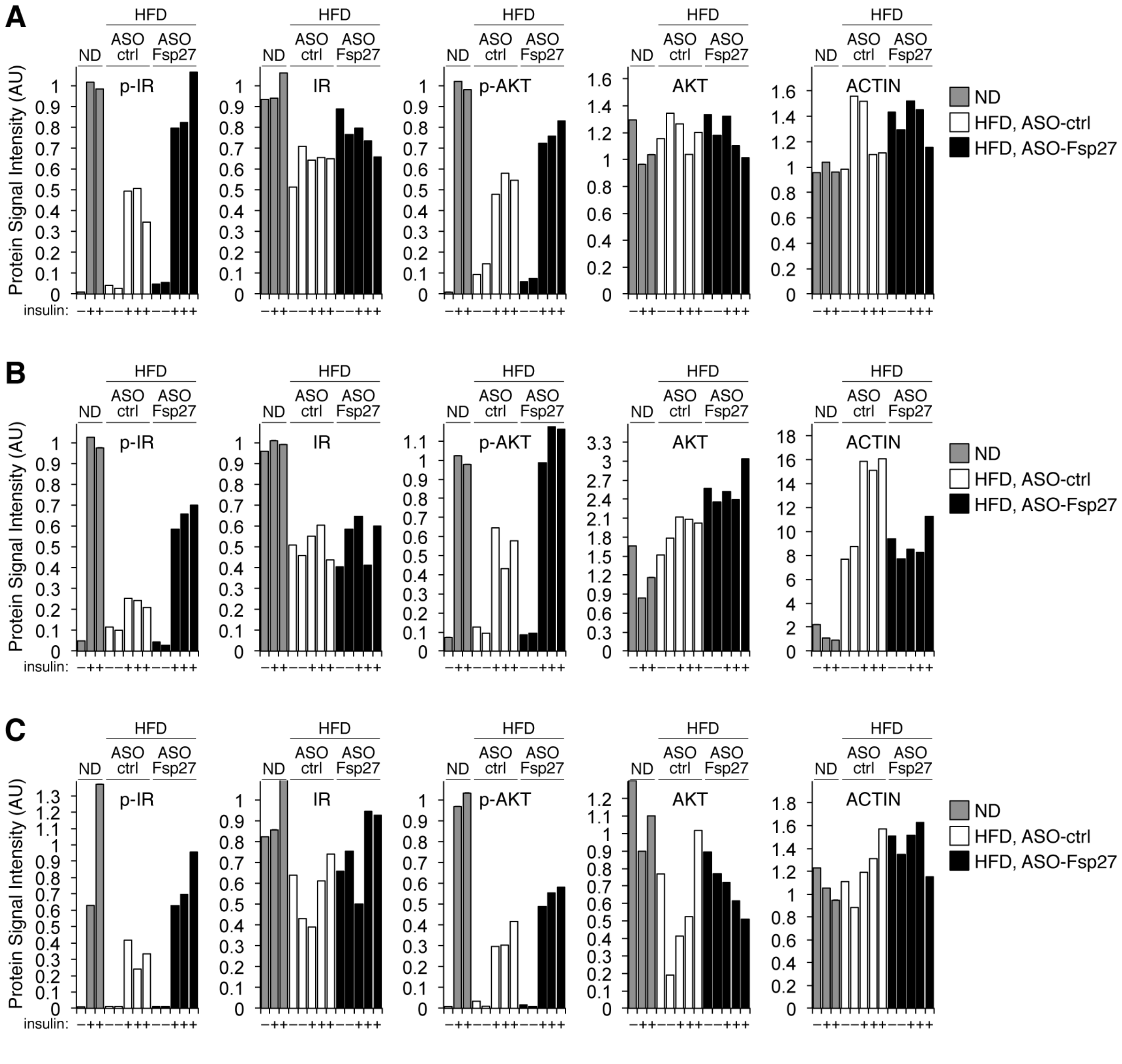


**Supplemental Figure S4.** Improved insulin sensitivity in mice treated with ASO-Fsp27. Quantification of immunoblots signal intensities shown in Fig. 5D–F.

| Transcript | Forward primer | Reverse primer |
|---|---|---|
| *36b4* | GGTGCCTCTGGAGATTTTCG | CACTGGTCTAGGACCCGAGAAG |
| *Acc* | TGACAGACTGATCGCAGAGAAAG | TGGAGAGCCCCACACACA |
| *Acox* | CAGCAGGAGAAATGGATGCA | GGGCGTAGGTGCCAATTATCT |
| *Adipoq* | GGAGAGAAAGGAGATGCAGGT | CTTTCCTGCCAGGGGTTC |
| *Atgl* | GCCTCCTTGGACACCTCAATAA | CTTCCTCGGGGTCTACCACA |
| *Cidea* | CTCCGAGTACTGGGCGATAC | ACCAGCCTTTGGTGCTAGG |
| *Cideb* | CTGCCAGCCTCCAAGAACT | TAGCACTCCACGTAGCAGCA |
| *Cox4* | TCACTGCGCTCGTTCTGAT | CGATCGAAAGTATGAGGGATG |
| *Cpt1a* | TGAGTGGCGTCCTCTTTGG | CAGCGAGTAGCGCATAGTCATG |
| *Cpt1b* | GAGTGACTGGTGGGAAGAATATG | GCTGCTTGCACATTTGTGTT |
| *Dio2* | CTGCGCTGTGTCTGGAAC | GGAGCATCTTCACCCAGTTT |
| *Hmgcr* | CTTGTGGAATGCCTTGTGATTG | AGCCGAAGCAGCACATGAT |
| *Hsl* | TTCTCCAAAGCACCTAGCCAA | TGTGGAAAACTAAGGGCTTGTTG |
| *Ldlr* | AGGCTGTGGGCTCCATAGG | TGCGGTCCAGGGTCATCT |
| *Fasn* | GCTGCGGAAACTTCAGGAAAT | AGAGACGTGTCACTCCTGGACTT |
| *Fsp27* | GGCTCACAGCTTGGAGGA | CTCCACGATTGTGCCATCT |
| *Mcad* | TTACCGAAGAGTTGGCGTATG | ATCTTCTGGCCGTTGATAACA |
| *Pcsk9* | GAAGACCGCTCCCCTGAT | GCACCCTGGATGCTGGTA |
| *Plin1* | GCTGCTTTCTCGGTGTTACAG | GAGCAGGTTCTCCTGCTCA |
| *Plin2* | CCTCAGCTCTCCTGTTAGGC | CACTACTGCTGCTGCCATTT |
| *Plin3* | CCACAGGATGCTGAAAAGG | TGATGTCCCTGAACATGCTG |
| *Plin4* | GGACTTACAAACAGCAACAGACC | TCTGTGAGTTGGTGGACACTTT |
| *Plin5* | ACATGGTGCTGGGCAAGT | TCAGCTGCCAGGACTGCTA |
| *Ppara* | CACCTGCAGAGCAACCATC | CCGAAGGTCCACCATTTTT |
| *Retn* | TTCCTTGTCCCTGAACTGCT | CCAATGTTCTTTATTGCATTTGG |
| *Scd1* | CCGGAGACCCCTTAGATCGA | TAGCCTGTAAAAGATTTCTGCAAACC |
| *Srebp1c* | GGAGCCATGGATTGCACATT | GGCCCGGGAAGTCACTGT |
| *Srebp2* | GCGTTCTGGAGACCATGGA | ACAAAGTTGCTCTGAAAACAAATCA |
| *Ucp1* | GGCCTCTACGACTCAGTCCA | TAAGCCGGCTGAGATCTTGT |

**Supplemental Table S1.** Oligonucleotides used in qPCR studies.
